# Masking the Close Eye Appearance in the East Asian Female Population: Infratemporal Hairline Reduction with Hair Grafting

**DOI:** 10.1007/s00266-016-0695-9

**Published:** 2016-09-08

**Authors:** Jae Hyun Park

**Affiliations:** Dana Plastic Surgery Clinic, Human Tower 11F, Gangnamdaero 605, Seocho-gu, Seoul, Korea

**Keywords:** Perceived close-set eyes, Infratemple area, Hairline, Hair transplantation

## Abstract

**Purpose:**

To investigate the effectiveness of hairline advancement in treating patients with perceived close-set eyes due to a wide infratemple area.

**Materials and Methods:**

Infratemple area hairline advancement was performed in 19 patients with perceived close-set eyes caused by a wide infratemple area; all were women with a mean age of 29.4 years.

**Results:**

The wide infratemple area was effectively narrowed in the frontal view in all patients. The mean reduction in the distance between the bilateral infratemple hairlines in the frontal view was 5.2 mm (range 3.9–6.3 mm). The appearance of close-set eyes was ameliorated, and patient satisfaction was high. No side effects, such as asymmetry or wound infection, were encountered.

**Conclusion:**

Hairline advancement surgery appears to be a safe and effective means of treating patients with perceived close-set eyes due to a wide infratemple area.

**Level of Evidence IV:**

This journal requires that authors assign a level of evidence to each article. For a full description of these Evidence-Based Medicine ratings, please refer to the Table of Contents or the online Instructions to Authors www.springer.com/00266.

## Introduction

The clinical definition of close-set eyes (CSE) is a distance between the medial canthi of less than the width of one eye. If the distance between the eyes is too narrow, the face may appear aesthetically displeasing. In these cases, lateral epicanthoplasty is generally performed or the lateral side of the eye may be extended using eyeliner cosmetics to create a lengthening effect.

**Fig. 1:**
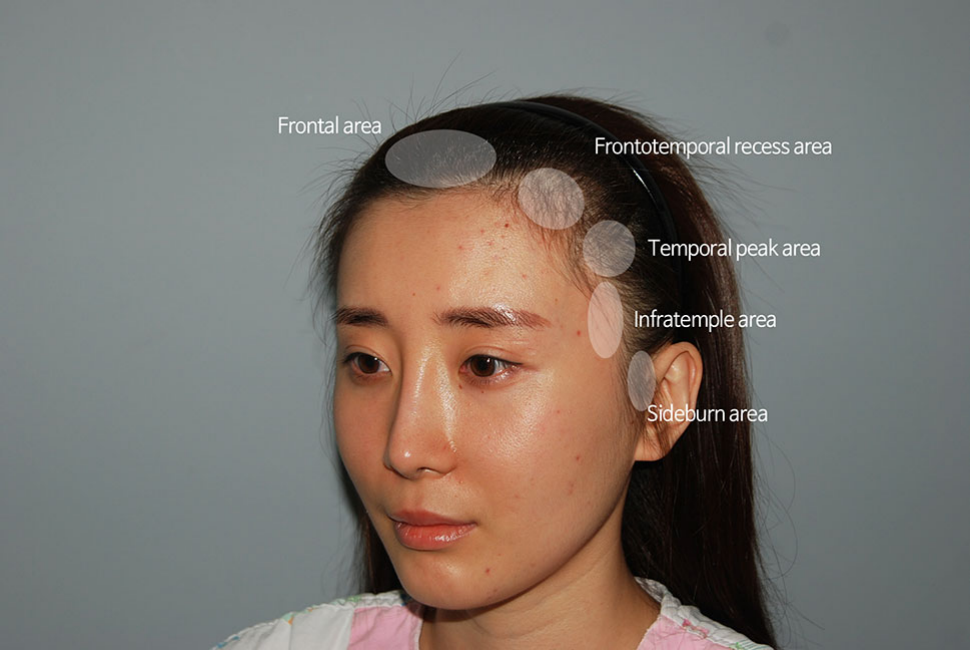
The infratemple area

It is not unusual for the intercanthal distance (ICD) to be greater than the width of one eye, and yet it nonetheless appears that the eyes are close together. In these cases, the infratemple area appears wider than normal when the face is viewed from the front. In these situations, the distance from the lateral canthus to the hairline of the infratemple area appears disproportionately great, and consequently the distance between the eyes appears relatively short. The infratemple area is located between the temporal peak and the sideburn area vertically, and acts as a bridge between the orbital cavity and the temporal bone in the horizontal plane (Fig. 1).

**Fig. 2:**
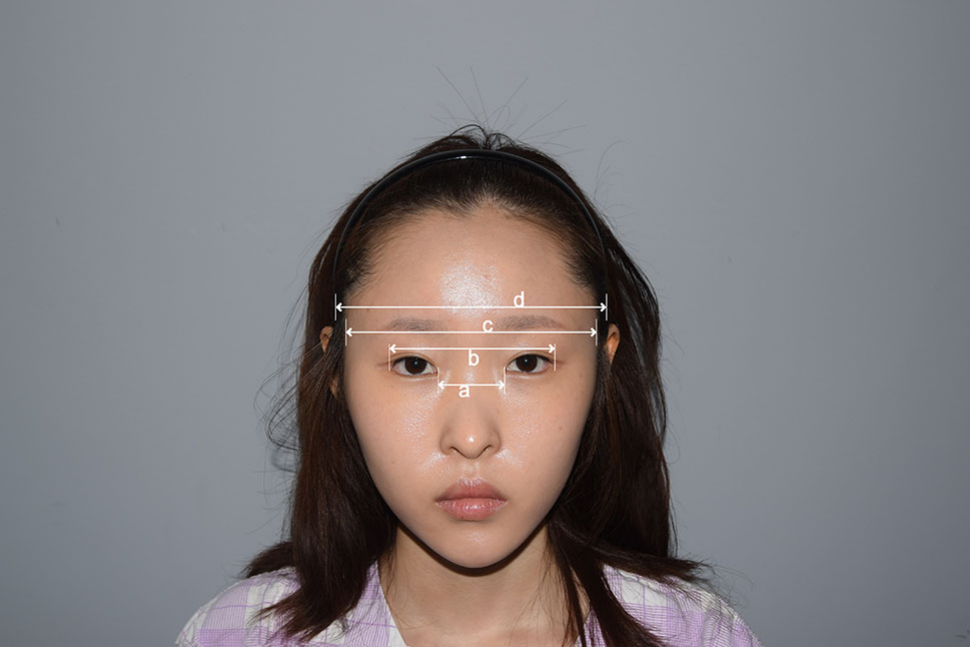
Anatomical landmarks for measurement from the frontal view: *a* intercanthal distance (ICD); *b* outercanthal distance (OCD); *c* distance between both infratemple area hairlines; *d* distance between the most laterally protruded points of the bilateral temporal bone

In cases in which the infratemple area is wide, the face appears flat or spread out horizontally, and may be considered unattractive [1]. Additionally, if this area is wide and the eyes appear relatively close together, patients may complain of CSE and feel unattractive, despite not fitting the clinical definition of CSE. The perception of CSE is magnified when patients wear their hair up in a ponytail, as their faces appear flat, with the eyes crowded together. Thus, patients avoid styling their hair in this manner, and may feel aesthetically challenged (Fig. 2).

Quant and colleagues reported that the mean ICD was 35.9 mm in Chinese men and 35.1 mm in Chinese women, compared with 31.7 and 30.8 mm in European Caucasian men and women, respectively [2]. Hwang and colleagues have reported that the mean ICD in Koreans is 35 mm ±standard deviation 3.3 mm [3], suggesting that—despite the potential for differences in measurement techniques between investigators—the normal ICD appears to be ≥30 mm in East Asian individuals [2, 3].

Consequently, we define individuals with an ICD <30 mm as having CSE. We define perceived CSE as an ICD >30 mm, in the presence of a difference between the bilateral infratemporal hairline distance and the outercanthal distance (OCD) that exceeds 50 mm (c–b in Fig. 2) and subjectively complaining about perceived-CSE appearance. This definition allows the ICD to be interpreted in the context of the face width. Individuals who do not fulfill any of these criteria yet still complain of CSE are considered to have pseudo-CSE. We examined the effectiveness of hairline advancement in the infratemple area in the treatment of perceived CSE.

## Materials and Methods

Medical charts and photos of patients who underwent infratemple area hairline advancement surgery due to perceived CSE were reviewed retrospectively by the author from 2013 to 2014.

We compared pre- and postoperative values of ICD (a), OCD (b), bilateral infratemple hairline distance (c), and bilateral temporal bone distances (d) (Fig. 2). A Vernier caliper (Mitutoyo, Kawasaki, Japan) was employed to ensure accurate and reproducible measurements in the frontal view.

The desired outcome was discussed with the patient before surgery while the patient was looking in a mirror. To advance the hairline of the infratemple area, an imaginary line was drawn from the T-point (the most anterior point of the temporal peak) and the S-point (the most anterior point where the sideburns begin). Then, a concave curved design, similar to the shape of the orbital rim, was made along this line. When drawing the design, the T-point was advanced if it was recessed. If the S-point was recessed or if the sideburns were sparse, the S-point was advanced with or without sideburn reconstruction to achieve sufficient narrowing of the infratemple area [4, 5].

Hair follicles to be used for transplantation were harvested with strip surgery of the occipital scalp or using the follicular unit extraction method. The harvested hairs were then classified into five subgroups: very thin, vellus-like single hairs, thin single hair, thick single hair, two-hair follicular units, and three- or four-hair follicular units.

In cases of transplantation to other areas, such as the frontotemporal recess, two- or three-hair follicular units were mostly transplanted in the posterior area, and only single hairs were transplanted in the infratemple area [6]. If insufficient single hairs were available, two- to three-hair follicular units were divided into single hairs.

In the infratemple area, thick single hairs were transplanted in the most posterior portion, with thin single hairs in front, and vellus-like and very thin single hairs in the most anteriorly located irregular peak area. Transplantation density was 35–55 FU/cm^2^ in the frontotemporal recess and 15–25 FU/cm^2^ in the infratemple and sideburn areas. Sutures were removed on the tenth postoperative day. Patients were examined 1 year after surgery. Satisfaction was scored on a Likert scale of 1 to 5 (1 very dissatisfied; 2 slightly dissatisfied; 3 neutral; 4 relatively satisfied; 5 very satisfied) by patients and the surgeon 1 year postoperatively.

## Results

**Fig. 3:**
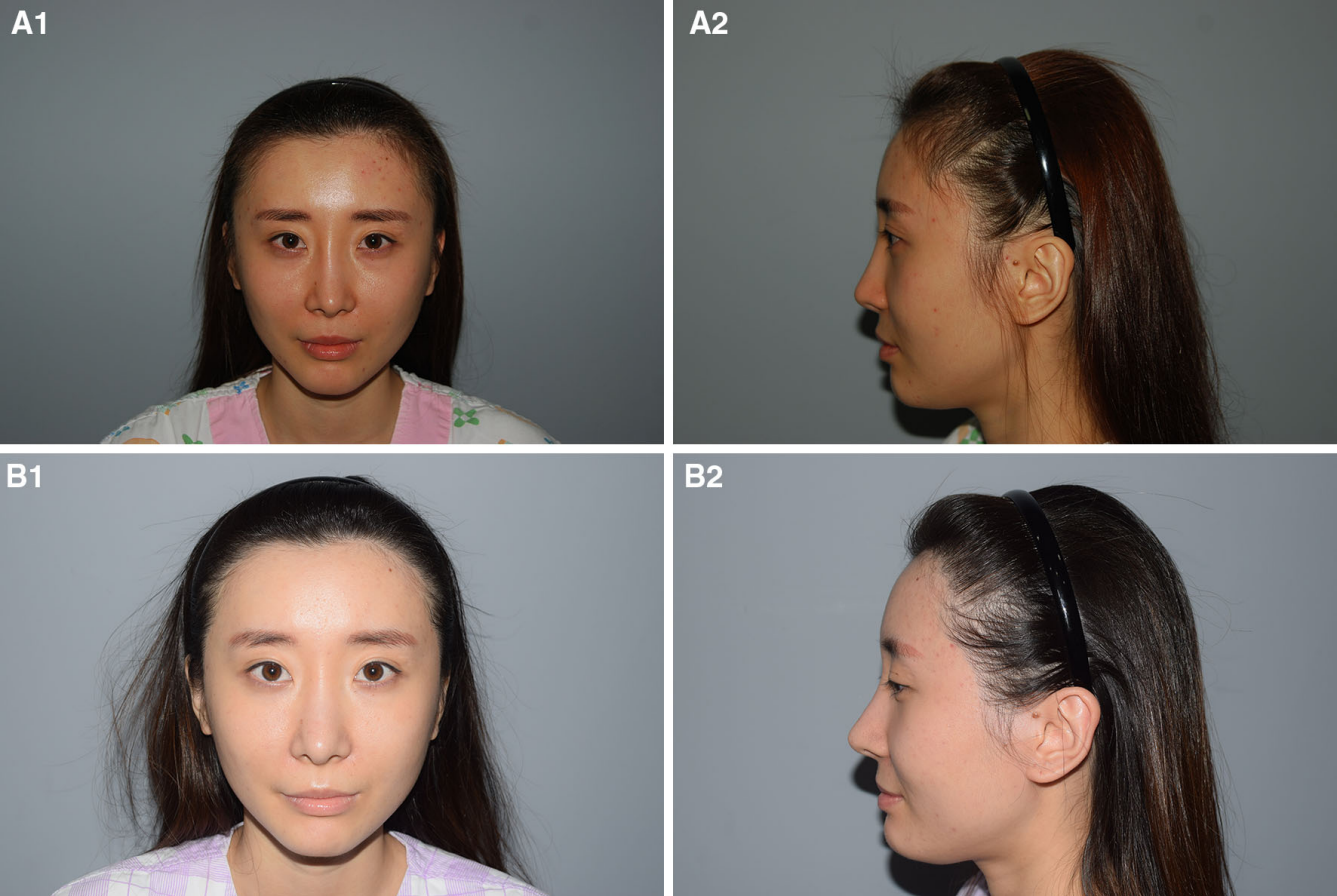
A 27-year-old woman with a CSE-like appearance due to a wide infratemple area. **a** Preoperative facial dimensions were (according to the scheme shown in Fig. 2): *a* 33; *b* 85; *c* 146; and *d* 164 mm. The infratemple hairline was recessed posteriorly with severe temporal bone lateral protrusion showing a broad bitemporal bone transverse distance. Infratemple hairline advancement with frontotemporal recess correction was undertaken, and 1190 hair grafts were transplanted. **b** Appearance 1 year after surgery. The *c* value improved to 140 mm. The patient stated that she no longer felt her eyes were too close together, and was satisfied with the results

Infratemple area hairline advancement was undertaken in 19 patients with perceived CSE. All patients were women, with a mean age of 29.4 years. The mean follow-up period was 12.3 months. In all patients, the wide infratemple area was effectively narrowed in the frontal view. On average, the distance between the bilateral infratemple area hairlines was reduced by 5.2 mm (3.9–6.3 mm, Table 1). The perception of CSE was also ameliorated, and the patients were satisfied with the results. The mean patient satisfaction score was 4.4, and the mean surgeon satisfaction score was 4.6. The satisfaction score was 4 or higher in all patients. No side effects, such as asymmetry or wound infection, were reported (Fig. 3).

**Table 1:** Facial dimensions of 19 patients with perceived CSE measured in the frontal view using a Vernier caliper

	Preoperative value	Postoperative value	Average difference
a (Intercanthal distance)	32 mm (30–34 mm)		
b (Outercanthal distance)	81 mm (79–86 mm)		
c (Bilateral infratemple hairline distance)	135 mm (130–141 mm)	129.8 mm (126–136 mm)	5.2 mm (3.2–6.4 mm)
d (Bilateral temporal bone distance)	154 mm (149–162 mm)		

Mean value, minimum value -maximum value indicated between parentheses

## Discussion

East Asians have a brachycephalic-type facial structure, which differs from Caucasians, who typically have dolichocephalic-type facial skeletons. Consequently, the transverse width of the face often appears greater in East Asians [1, 4]. Cosmetic procedures have been developed in an attempt to make the face look slimmer [4, 5, 7]. Unlike for Caucasians, who do not appear to have a wide face in the frontal view, hairline correction of the infratemple area has the potential to yield clinically significant improvements in perceived facial appearance for East Asians [1, 4]. Other than the author’s own studies, there is little evidence about the efficacy of female hairline correction surgery using hair transplantation in the published literature.

**Fig. 4:**
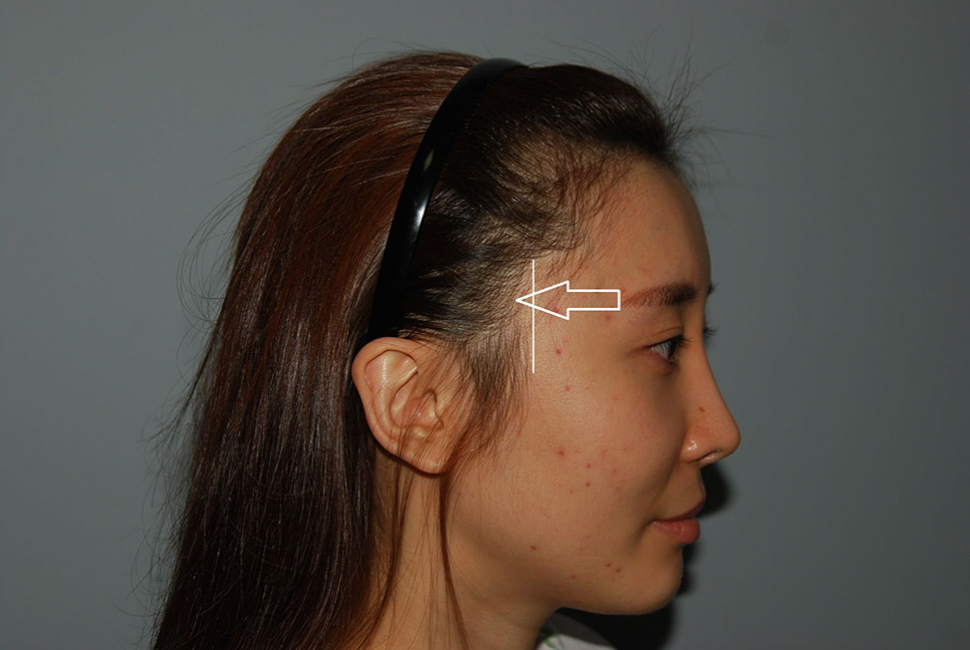
The infratemple area hairline has been recessed posteriorly from the vertical plane starting from the most anterior point of the sideburn

**Fig. 5:**
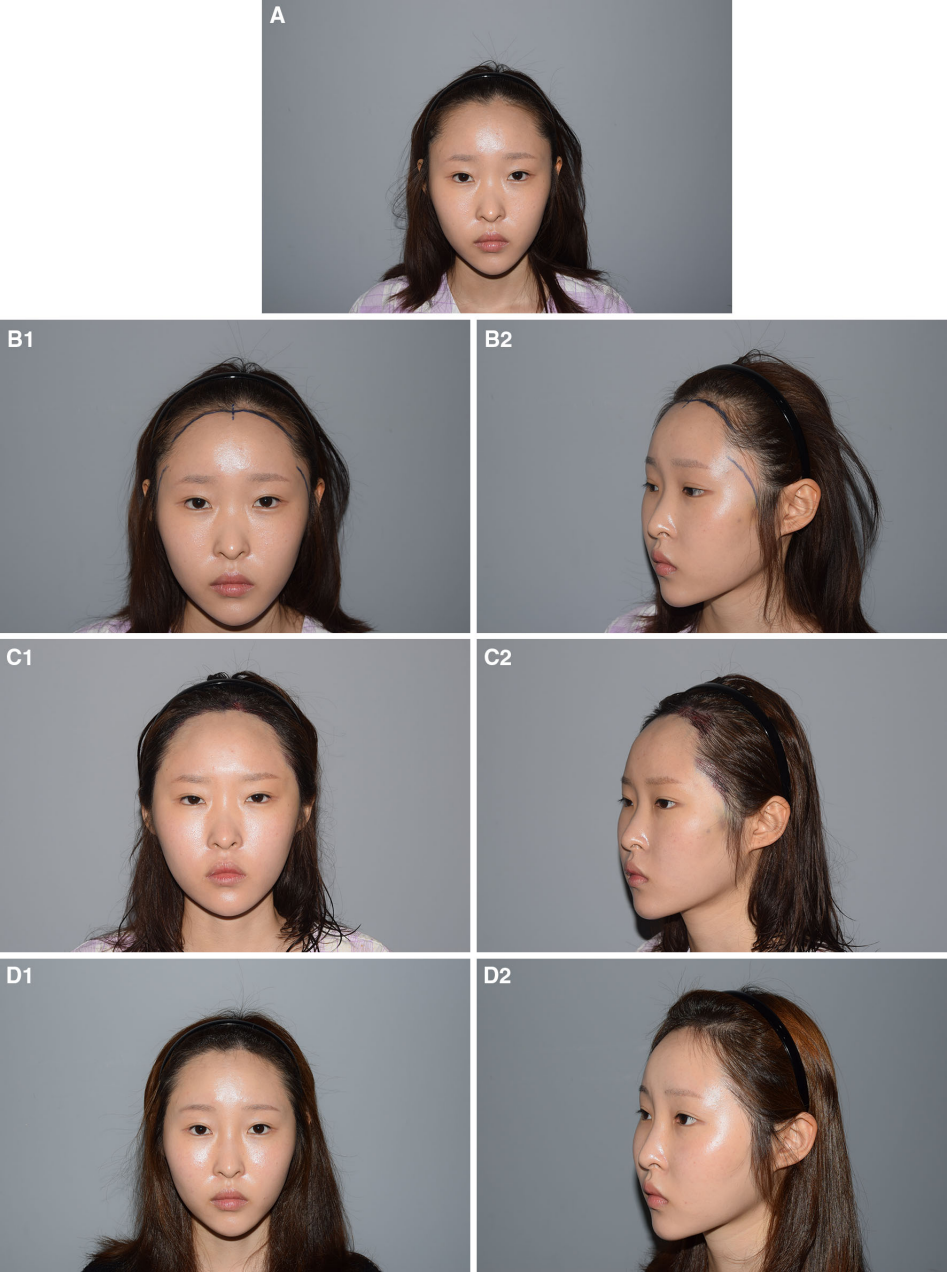
A patient in whom the distance between the lateral canthi is small (pseudo-CSE). This 23-year-old woman has eyes with a short transverse width; so, although the distance between the medial canthi was normal, the distance between the lateral canthi appeared relatively short due to a wide infratemple area. The patient had undergone lateral canthoplasty in the past, but the effect had been minimal. Preoperative facial dimensions were (according to the scheme shown in Fig. 2): *a* 32 mm; *b* 81 mm; *c* 135 mm; *d* 154 mm. The c–b difference was 54 mm **a** preoperative design. **b** immediate postoperative view with some postoperative edema in the recipient area. **c** facial appearance 12 months postoperatively: the bilateral infratemple distance (*c*) was reduced to 129 mm. The pseudo-CSE appearance was ameliorated, and the patient was highly satisfied with the results

Park reported that zygoma reduction or hairline correction surgery can be performed to narrow the width of the midface, and the shape of the zygomatic bone can be an important factor in selecting the type of procedure [4]. Also according to Park, it is sometimes effective to advance the anterior starting point of the sideburns anteriorly to narrow the midface [5]. The location of the infratemple area between the temporal bone and the orbit means that it is not greatly affected by the shape of the zygomatic arch. There are situations in which the infratemple area is widened, e.g., when there is recession of the infratemporal hairline, lateral protrusion of the temporal bone, and a short OCD [1]. These causes may exist separately or together (Figs. 3, 4, and 5).

Only single follicular unit hair should be used for side-hairline correction [4, 6]. Unless the follicles are carefully transplanted with matched angles and directions, the final results may appear awkward and unnatural. It is important to transplant the hair follicles with care so that they match the direction, angle, and thickness of existing hairs. Furthermore, an acute angle must be maintained in this area. A horizontal direction should be adopted at the T-point, and this must become increasingly vertical when approaching the S-point. If the hairline is advanced too far, it may become too close to the eyebrows, which is also aesthetically awkward. A distance of at least 2 cm should be maintained from the eyebrow.

This study had some limitations. There was a relatively small number of cases, and no comparison was made with other surgical strategies such as lateral canthoplasty; we intend to address these limitations in future studies.

## Conclusion

Hairline advancement appears to be an effective treatment for perceived CSE caused by a wide infratemple area.
